# Facial Skin Care Instruction by Medical Professionals Using Microneedle Cream: The 8‐Week Prospective, Randomized, Single‐Blinded for Examiners Trial in Asians

**DOI:** 10.1111/jocd.70680

**Published:** 2026-02-02

**Authors:** Toko Mori, Yurika Osuji, Ruriko Kawanabe, Takemichi Fukasawa, Jun Omatsu, Asako Yoshizaki, Takashi Yamashita

**Affiliations:** ^1^ Department of Dermatology University of Tokyo Graduate School of Medicine Tokyo Japan

**Keywords:** melanin, microneedle cream, skin care instruction, skin moisturization, skin viscoelasticity

## Abstract

**Background:**

Skin care is widely regarded as a fundamental component of self‐care. However, few studies have systematically evaluated the effects of structured skin care instruction on both objective parameters and subjective perceptions. This study aims to elucidate these effects through a controlled clinical investigation.

**Methods:**

This clinical trial was conducted at the University of Tokyo Hospital between July and September 2023. Twenty‐one healthy women aged 30–60 years applied a microneedle cream to the face for 8 weeks. They were randomly assigned to either the intervention group, which received skin care instructions from the start, or the control group, which received them after 4 weeks. Skin hydration, transepidermal water loss, viscoelasticity, and melanin content were assessed at baseline, week 4, and week 8. Subjective evaluations were obtained via structured questionnaires. Statistical analyses were conducted using Prism (version 10.2.3).

**Results:**

At week 4, the intervention group showed significantly greater improvements in skin hydration (intracorneal water content: −0.98% ± 13.49% [control] vs. 14.06% ± 14.61% [intervention], *p* < 0.05) and elasticity (R7: −3.48% ± 10.36% [control] vs. 7.92% ± 10.29% [intervention], *p* < 0.05) compared to the control group. At week 8, melanin pigmentation was significantly reduced in the intervention group (Mark‐Vu: 11.44% ± 26.67% [control] vs. −15.01% ± 11.10% [intervention], *p* < 0.05). Subjective assessments were consistent with these objective results, reflecting improved skin perception.

**Conclusions:**

This is the first study to demonstrate that appropriate skin care practices can lead to the moisturizing, firming, and elasticity effects of a microneedle cream. These findings highlight the importance of proper product use in maximizing the efficacy of skin care interventions.

**Trial Registration:**

UMIN: UMIN000052080

## 1 Introduction

With aging, human skin becomes drier, less firm, and more prone to sagging. Wrinkles deepen, pigmentation such as age spots appears, and structural changes in facial contours become increasingly evident [1, 2]. Anti‐aging strategies aim to mitigate these effects and play a vital role in improving quality of life (QOL). The American Academy of Dermatology (AAD) recommends several skin care practices for anti‐aging, including sun protection, avoiding artificial tanning, daily moisturizing, washing the face twice daily, abstaining from smoking, maintaining a healthy diet, and ensuring adequate sleep [3]. Among these, proper skin care is a fundamental component, contributing to the prevention of hyperpigmentation, maintenance of skin firmness, reduction of fine lines and wrinkles, enhancement of skin radiance, prevention of photoaged skin, and reduction in skin cancer risk [3, 4].

Evidence indicates that proper skin care can prevent xerosis (dry skin) [5, 6]. For instance, atopic dermatitis, rosacea, and acne, intraepidermal water content is decreased and transepidermal water loss (TEWL) is elevated [7, 8, 9, 10]; however, these parameters have been demonstrated to improve with appropriate skin care [7]. Despite its routine nature, a gap often exists between healthcare professionals and patients in the understanding and execution of skin care techniques. Various strategies have been proposed to bridge this gap. One such example is the “finger‐tip unit” used in the management of chronic skin diseases, which standardizes the amount of topical medication applied and improves treatment outcomes by ensuring consistent dosage and technique [11]. Studies have demonstrated that educating patients on appropriate application volume and method significantly enhances therapeutic efficacy [12, 13, 14, 15]. In contrast, daily anti‐aging skin care is often self‐administered and may vary considerably among individuals, potentially affecting its efficacy. While several studies have demonstrated improvements in objective indicators following hand skin care instruction [13, 16, 17], no studies to date have systematically evaluated the effects of facial skin care instruction, potentially relevant to anti‐aging, on both subjective and objective parameters.

We hypothesize that minor variations in daily skin care practices can lead to significant differences in anti‐aging efficacy. This study focuses on women aged 30 years and older and investigates the effects of structured skin care guidance using a microneedle cream formulation. A blinded study was conducted to assess changes in physiological skin parameters, including intraepidermal water content, and to evaluate the impact of skin care guidance on both skin condition and QOL.

## 2 Materials and Methods

### 2.1 Study Design and Patient Enrollment

This prospective, examiner‐blinded study was conducted at the University of Tokyo Hospital (Bunkyo‐ku, Tokyo, Japan) from July to September 2023, and included 21 healthy Asian women aged 30 to 60 years. Major exclusion criteria were: a history of hypersensitivity or contact dermatitis to any of the test ingredients or similar compounds; active dermatoses at the application site; pregnancy or lactation; malignancy; and a history of facial surgery or cosmetic procedures within 4 weeks prior to study initiation. All participants provided both verbal and written informed consent. The study was conducted in accordance with the Declaration of Helsinki, approved by the Research Ethics Committee of the Faculty of Medicine, University of Tokyo (approval number: 2023011NI).

### 2.2 Microneedle Cream

The microneedle cream used in this study (Hakase Beaute Permeative Premium PRO Cream, YA‐MAN Ltd., Japan) contains a high concentration of microneedles composed of hydrophilic biomacromolecules derived from marine sponge spicules. These microneedles exhibit a needle‐like shape and measure approximately 200 μm in length, 15 μm in width, and 2 μm at the tip. As detailed in Table S1, the formulation contains sodium hyaluronate, hydrolyzed collagen, niacinamide, and additional botanical ingredients. Upon application, the microneedles adhere to the skin and create microscopic pores in the stratum corneum, facilitating the penetration of active ingredients into the epidermis [18, 19].

### 2.3 Intervention

Stratified block randomization was used to allocate 21 female participants into two groups: the “control” group (*n* = 10), which received skin care instruction at week 4 only, and the “intervention” group (*n* = 11), which received the skin care instruction at both baseline and week 4. Participants were instructed to apply the microneedle cream within 5 min after washing their face, twice daily, without using lotion or emulsion. The skin care instruction was as follows: (1) applying a pearl‐sized amount of cream gently across the face, starting with the cheeks, perioral area, and periorbital area; (2) tapping lightly around the eyes and mouth; (3) layering the cream over areas of concern; and (4) blending the cream over the face using the entire palm. This instruction was presented via a 2‐min instructional video followed by a detailed explanation using an instructional sheet (Table S2), which participants were allowed to take home. During the study period, the participants were instructed to avoid excessive UV exposure (e.g., swimming, mountain climbing, sunbathing, and outdoor exercise), to use UV protection, and to refrain from initiating new supplement use. To eliminate confounding effects from other skin care products, the use of strongly moisturizing or anti‐wrinkle cosmetics, drugs, or quasi‐drugs on the face was prohibited from one week prior to the study. No restrictions were placed on cleansers, face washes, sunscreens, and commonly used makeup products. All skin care guidance was provided by board‐certified dermatologists who were not involved in outcome assessments.

### 2.4 Efficacy Evaluation

The following evaluations were conducted at three time points: before application (baseline), and at weeks 4 and 8 after the start of the treatment. Prior to each evaluation, the facial skin was thoroughly cleansed and allowed to acclimate for 20 min. The primary outcome was the change in intraepidermal water content, measured using a physiological assessment device. Secondary outcomes included TEWL, skin elasticity, erythema, melanin pigmentation, and self‐assessed skin condition and QOL. Stratum corneum hydration, TEWL, elasticity, erythema, and melanin pigmentation were quantitatively measured using specialized instruments. Subjective skin condition and QOL were evaluated through a structured, scored self‐assessment questionnaire.

### 2.5 Device‐Based Evaluation

The measurement time points were predefined as baseline (prior to the initiation of application), week 4, and week 8. Before each measurement, the left cheek was thoroughly cleansed and acclimatized for 20 min. Subsequently, the defined area on the left cheek was measured repeatedly. The measurement of intracorneal water content was conducted five times using the Corneometer CM825 (Courage + Khazaka Electronic GmbH, Cologne, Germany). Additionally, TEWL was measured twice using the Tewameter TM HEX (Courage + Khazaka Electronic GmbH, Cologne, Germany) [16, 20]. Skin firmness and elasticity were measured in the same area using the Cutometer DUAL MPA580 (Courage + Khazaka Electronic GmbH, Cologne, Germany) equipped with 2 and 6 mm probes [20, 21, 22]. Each measurement was performed for 5 s under a pressure of 400 mbar and repeated five times. Two apparent outliers were excluded, and the remaining three measurements were used for analysis. The skin's resistance to negative pressure (stiffness) and its ability to return to its original position (elasticity) are displayed as real‐time measurement curves (penetration depth in mm). The parameters considered include R2, which represents viscoelasticity (the ratio of skin resistance during suction to the ability to return during relaxation), R5, which defines net elasticity (the ratio of the elastic portion during suction to elastic portion after relaxation), and R7, which is the ratio of elastic return to the overall deformation. Furthermore, for melanin assessment, the Mexameter MX18 (Courage + Khazaka Electronic GmbH, Cologne, Germany) is the instrument of choice. These instruments have been described in scientific publications and have received numerous citations, making them suitable for this study.

Melanin levels were additionally assessed using the Mexameter MX18 (Courage + Khazaka Electronic GmbH, Cologne, Germany) [20, 22]. As with the Cutometer measurements, five readings were obtained for each subject, and two apparent outliers were excluded. The mean of the remaining three values was used for analysis. These instruments have been widely used and validated in peer‐reviewed scientific literature and have received numerous citations, supporting their appropriateness for use in this study [20, 21, 22]. Further, the assessment of melanin levels, erythema, and future wrinkles formation was performed using the Mark‐Vu system (PSI PLUS Co., Korea), a diagnostic skin imaging device commercially known in Japan as “neo voir”. This instrument offers multiple imaging modes, including normal, ultraviolet (UV), polarized, and gloss. The machine is equipped with four distinct LED light sources [23]. It enables high‐resolution visualization of various skin characteristics, such as pores, wrinkles, redness, dullness, skin tone, and porphyrins. For each respective outcome, melanin content was evaluated under UV mode, erythema was evaluated under polarized mode, and future wrinkles were measured using gloss mode. To ensure consistency across images, brightness levels were adjusted using the system's dedicated analysis application, and focus was also standardized by photographing a calibration chart including with the equipment. The analysis application was developed based on a reference database of 3000 Korean individuals, and its validity has been established through comparative analyses using this dataset [24]. In preparation for the clinical trial, evaluators underwent a comprehensive three‐month training program covering all measurement protocols and device handling. All assessments were carried out by multiple board‐certified dermatologists who were blinded to group allocation. This procedural separation was implemented to maintain assessment objectivity and minimize potential evaluator bias throughout the study.

### 2.6 Self‐Evaluation Questionnaires

Participants were asked to respond to eight questions regarding skin condition using a 9‐point scale (1: worst, 9: best) at three time points: baseline, week 4, and week 8. They were also instructed to evaluate changes in their skin condition by comparing scores across these time points. The 8 evaluated items included skin texture, skin moisturization, firmness, tightening, crow's feet, mouth frown, brown spots, and pores. In addition to these self‐assessments, the Skindex‐16, a globally recognized QOL assessment scale, was utilized. The scale consists of 16 items divided into three subscales (Symptoms, Emotions, and Function). Participants rated how much specific skin‐related problems had bothered them during the past week using a 7‐point scale (0: never bothered to 6: always bothered) [25].

### 2.7 Safety Assessments

A dermatological evaluation was conducted at each assessment point to examine the presence of erythema, dryness, pruritus, edema, urticaria, and other relevant cutaneous manifestations. In addition, a daily logbook was distributed to all participants prior to the initiation of the study, and they were instructed to record any adverse events and subjective symptoms experienced during the study period.

### 2.8 Statistical Analysis

The study included a total of 18 participants. Within‐group comparisons were conducted using one‐way ANOVA followed by Tukey's post hoc test (the Friedman test with Dunn's multiple comparisons was applied for melanin measurements obtained via the Mexameter in the intervention group). Between‐group comparisons were performed using unpaired *t*‐tests. For Skindex‐16 scores, the Wilcoxon signed‐rank test was used. A two‐tailed *p*‐value of < 0.05 was considered statistically significant. Values are presented as mean ± standard deviation unless otherwise specified. Statistical analyses were performed using Prism version 10.2.3 (GraphPad Software, San Diego, CA, USA).

## 3 Results

### 3.1 Participants

A total of 21 participants were enrolled in the study. Two of them withdrew due to subjective symptoms, such as pruritus and irritation, and one withdrew due to scheduling conflicts. Among the 18 participants who completed the study, the mean age was 43.94 ± 7.81 years (range 31–54). Of these, 9 were allocated to the control group and 9 to the intervention group. Table 1 presents the characteristics of the participants.

**TABLE 1:** Demographic characterization of the patients.

Control group				Intervention group			
	Age	Sex	Race		Age	Sex	Race
1	51	F	Asian	1	39	F	Asian
2	52	F	Asian	2	48	F	Asian
3	32	F	Asian	3	51	F	Asian
4	36	F	Asian	4	50	F	Asian
5	36	F	Asian	5	54	F	Asian
6	41	F	Asian	6	49	F	Asian
7	44	F	Asian	7	31	F	Asian
8	34	F	Asian	8	38	F	Asian
9	51	F	Asian	9	54	F	Asian
10	43	F	Asian	10	46	F	Asian
11	43	F	Asian				
	Mean ± SD				Mean ± SD		
Age	42.1 ± 6.78			Age	46 ± 7.21		(*p* = 0.2381)

*Note:* A total of 21 healthy Asian women, aged between 30 and 60 years, were randomly assigned to either the control group or the intervention group. This table presents the age, sex, and race of all enrolled participants, including those who withdrew from the study. The median age for each group is reported, providing a statistical summary of the age distribution within each cohort.

### 3.2 Improvement of Skin Moisturization in the Intervention Group

**FIGURE 1:**
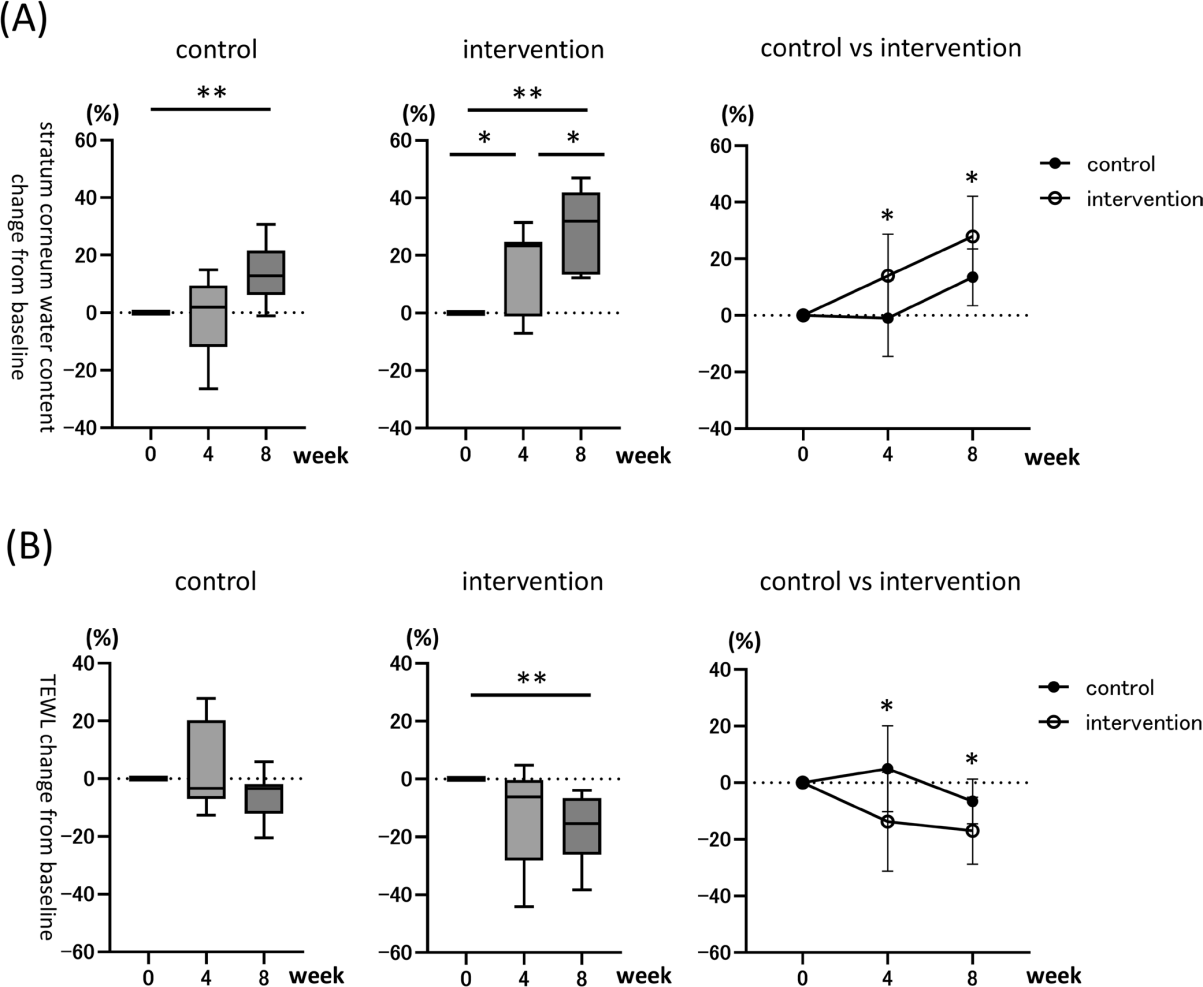
Changes in skin hydration. (A) Change in intracorneal water content assessed by the Corneometer at baseline, week 4, and week 8 in both the control and intervention groups. Left and middle panels: Within‐group comparisons of intracorneal water content at baseline, week 4, and week 8 (control group: *N* = 9; intervention group: *N* = 9). Box plots represent the median (line within the box), the interquartile range (IQR; box), and the whiskers indicate the minimum and maximum values. *p < 0.05, **p < 0.01 vs. baseline, week 4, or week 8, as applicable. Right panel: Between‐group comparisons of intracorneal water content at weeks 4 and 8 (*n* = 9 per group). Error bars indicate SD. *p < 0.05 for comparisons between the control and intervention groups. (B) Change in TEWL assessed by the Tewameter at baseline, week 4, and week 8 in both the control and intervention groups. Left and middle panels: Within‐group comparisons of TEWL at baseline, week 4, and week 8 (*n* = 9 per group). Box plots represent the median (line within the box), the interquartile range (IQR; box), and the whiskers indicate the minimum and maximum values. *p < 0.05, **p < 0.01 vs. baseline, week 4, or week 8, as applicable. Right panel: Between‐group comparisons of TEWL at weeks 4 and 8 (*n* = 9 per group). Error bars indicate SD. *p < 0.05 for comparisons between the control and intervention groups.

The Corneometer CM825 was used to assess stratum corneum water content [20], with percentage changes calculated at weeks 4 and 8 for both the control and intervention groups relative to baseline (Figure 1A, left and middle panel). An increase in intracorneal water content was observed in both groups throughout the study. In the control group, a significant increase was detected at week 8 (13.52% ± 10.04%, *p* < 0.01), whereas no significant change was observed at week 4. In contrast, the intervention group exhibited a significant improvement as early as week 4 (14.06% ± 14.61%, *p* < 0.05), which further increased at week 8 (27.99% ± 14.18%, *p* < 0.01). Between‐group comparisons revealed a significant difference in water content at both weeks 4 and 8 (Figure 1A, right panel, 4‐week: −0.98% ± 13.49% [control] vs. 14.06% ± 14.61% [intervention]; 8‐week: 13.52% ± 10.04% [control] vs. 27.99% ± 14.18% [intervention], *p* < 0.05). TEWL was assessed using the Tewameter TM HEX, which measures the density gradient of water vapor flux from the skin surface [16, 20]. Changes in TEWL at weeks 4 and 8 were calculated relative to baseline values (Figure 1B). In the control group, TEWL exhibited a decreasing trend at week 8; however, the change was not statistically significant (Figure 1B, left panel, −6.58% ± 7.87%, *p* = 0.08). In the intervention group, a significant reduction in TEWL was observed at week 8 compared to baseline (Figure 1B, middle panel, −16.95% ± 11.87%, *p* < 0.01). Between‐group comparisons showed a significantly greater reduction in TEWL in the intervention group at both weeks 4 and 8 (Figure 1B, right panel, 4‐week: 4.97% ± 15.15% [control] vs. −13.75% ± 17.43% [intervention], *p* < 0.05; 8‐week: −6.58% ± 7.87% [control] vs. −16.95% ± 11.87% [intervention], *p* < 0.05).

### 3.3 Improvement of Viscoelasticity From the Epidermis to the Deep Dermis in the Intervention Group

**FIGURE 2:**
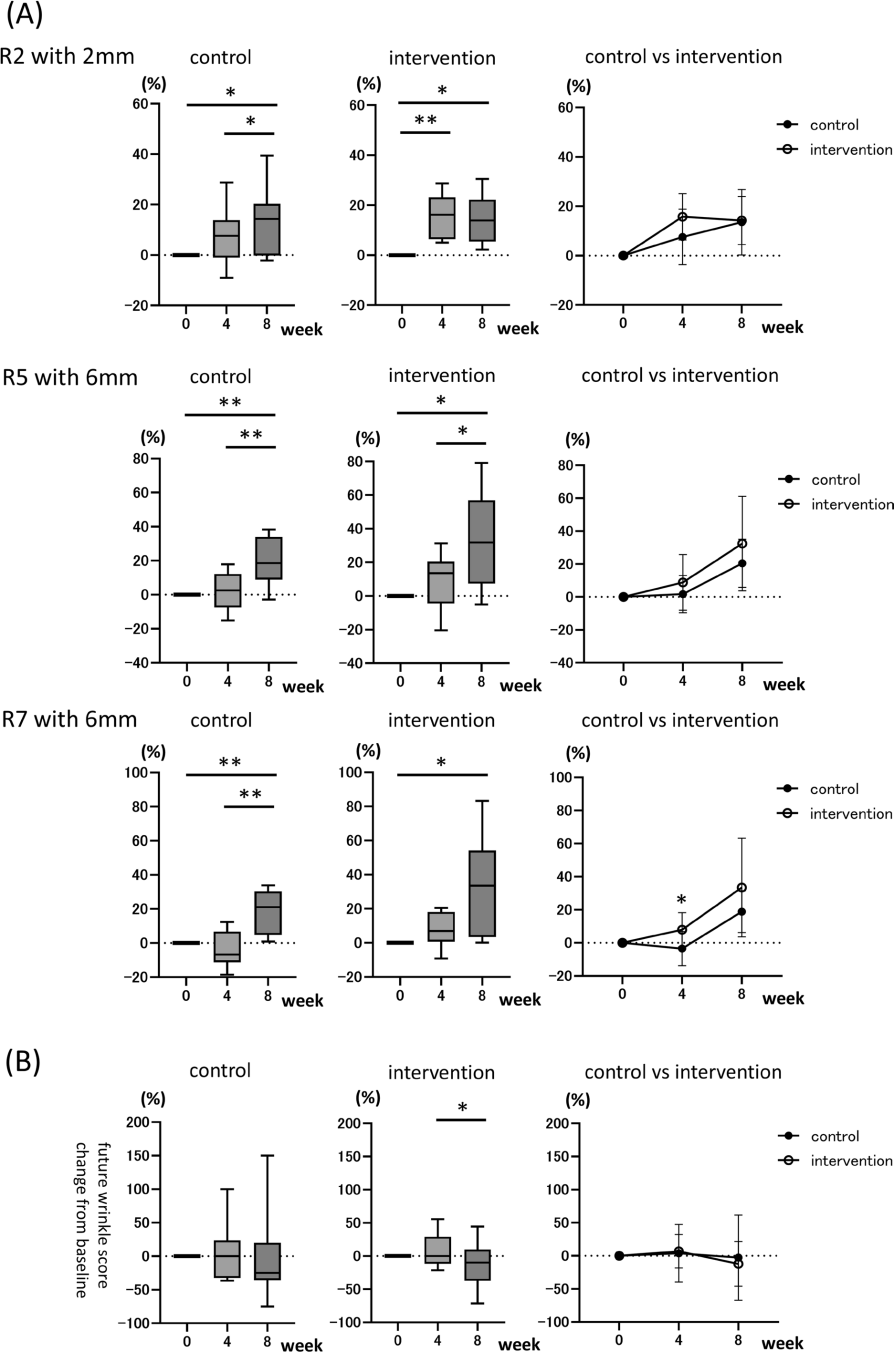
Changes in skin viscoelasticity. (A) Changes in viscoelasticity and elasticity measured using the Cutometer. Measurements were taken at baseline, week 4, and week 8 in both the control group (*n* = 9) and intervention group (*n* = 8). The first row: Change in R2 at 2 mm‐probe setting. Left and middle panels: Within‐group comparisons at baseline, week 4, and week 8 for the control and intervention groups. Box plots represent the median (line within the box), the interquartile range (IQR; box), and the whiskers indicate the minimum and maximum values. *p < 0.05, **p < 0.01 vs. baseline, week 4, or week 8 as applicable. Right panel: Between‐group comparisons at weeks 4 and 8. Error bars represent SD. *p < 0.05 vs. control. The second row: Change in R5 at 6 mm‐probe setting. Left panel middle panels: Within‐group comparisons at baseline, weeks 4 and 8 for the control and intervention groups. Box plots represent the median (line within the box), the interquartile range (IQR; box), and the whiskers indicate the minimum and maximum values. *p < 0.05, **p < 0.01 vs. baseline, week 4, or week 8 as applicable. Right panel: Between‐group comparisons at weeks 4 and 8. Error bars represent SD. *p < 0.05 vs. control. The third row: Change in R7 at 6 mm‐probe setting. Left panel and middle panels: Within‐group comparisons at baseline, weeks 4 and 8 for the control and intervention groups. Box plots represent the median (line within the box), the interquartile range (IQR; box), and the whiskers indicate the minimum and maximum values. *p < 0.05, **p < 0.01 vs. baseline, week 4, or week 8 as applicable. Right panel: Between‐group comparisons at weeks 4 and 8. Error bars represent SD. *p < 0.05 vs. control. (B) Changes in predicted future wrinkles measured using Mark‐Vu system, comparing baseline, week 4 and week 8 in both the control and intervention groups (*n* = 9 per group). Left and middle panels: Within‐group comparisons at baseline, week 4, and week 8. Box plots represent the median (line within the box), the interquartile range (IQR; box), and the whiskers indicate the minimum and maximum values. *p < 0.05 compared with baseline, week 4, or week 8, as applicable. Right panel: Comparison between the control and intervention groups at weeks 4 and 8 (*n* = 9 per group). Error bars represent SD. *p < 0.05 vs. control.

The viscoelasticity of the skin was measured using a Cutometer DUAL MPA580 [20, 21, 22]. R2 indicates viscoelasticity, R5 indicates net elasticity (excluding viscous effects), and R7 indicates elasticity, as previously described [21]. The 2‐mm probe of the Cutometer reflects properties from the epidermis to the superficial dermis, while the 6‐mm probe reflects those from the superficial to the deep dermis. R2 with the 2‐mm probe was used to measure viscoelasticity, and R5 and R7, with the 6‐mm probe, were used to measure elasticity. Changes from baseline were measured at weeks 4 and 8 (Figure 2A). For R2, both the control and intervention groups exhibited increased recovery from deformation at weeks 4 and 8 compared to baseline (Figure 2A, the first row, left and middle panels). In the control group, no significant difference was observed at week 4; however, a significant increase was observed at week 8 (13.54% ± 13.24%, *p* < 0.05), as well as between weeks 4 and 8 (7.60% ± 11.26% [4‐week] vs. 13.54% ± 13.24% [8‐week], *p* < 0.05). In the intervention group, a significant increase was already evident at week 4 (15.75% ± 9.37%, *p* < 0.01) and was sustained through week 8 (14.23% ± 9.75%, *p* < 0.05). Between‐group comparisons revealed the greatest difference at week 4, which narrowed by week 8 (Figure 2A, the first row, right panel). For R5, both groups exhibited increased elasticity compared with the baseline (Figure 2A, the second row, left and middle panels). In the control group, no significant change was observed at week 4, while a significant increase was evident at week 8 (20.48% ± 14.58% [8‐week], *p* < 0.01; 1.76% ± 11.33% [4‐week] vs. 20.48% ± 14.58% [8‐week], *p* < 0.01). The intervention group showed a slight increase at week 4, followed by a more substantial improvement at week 8 (8.87% ± 16.92% [4‐week] vs. 32.49% ± 28.71% [8‐week], *p* < 0.05). A significant increase was also observed compared with baseline (32.49% ± 28.71% [8‐week], *p* < 0.05). However, between‐group differences were not statistically significant through week 8 (Figure 2A, second row, right panel). For R7, both groups demonstrated increased elasticity relative to the baseline (Figure 2A, the third row, left and middle panels). In the control group, elasticity slightly declined at week 4 and then significantly improved at week 8 (compared with baseline, 18.87% ± 12.66% [8‐week], *p* < 0.01; compared with 4 weeks, −3.48% ± 10.36% [4‐week] vs. 18.87% ± 12.66% [8‐week], *p* < 0.01). In the intervention group, a slight increase was observed at week 4, followed by a significant improvement at week 8 (33.42% ± 29.79% [8‐week], *p* < 0.05). A comparison between the two groups showed significant differences at week 4 (Figure 2A, the third row, right panel, −3.48% ± 10.36% [control] vs. 7.92% ± 10.29% [intervention], *p* < 0.05).

Evaluation of future wrinkles using the Mark‐Vu system [23, 24] corroborated these findings (Figure 2B). The analysis revealed a reduction in future wrinkles by week 8 in both groups (Figure 2B, left panel). In the intervention group, future wrinkle scores decreased significantly from week 4 to week 8 (Figure 2B, middle panel, 6.99% ± 25.44% [4‐week] vs. −12.12% ± 33.84% [8‐week], *p* < 0.05). There were no significant differences between the two groups (Figure 2B, right panel).

### 3.4 Melanin Changes in the Intervention Group

**FIGURE 3:**
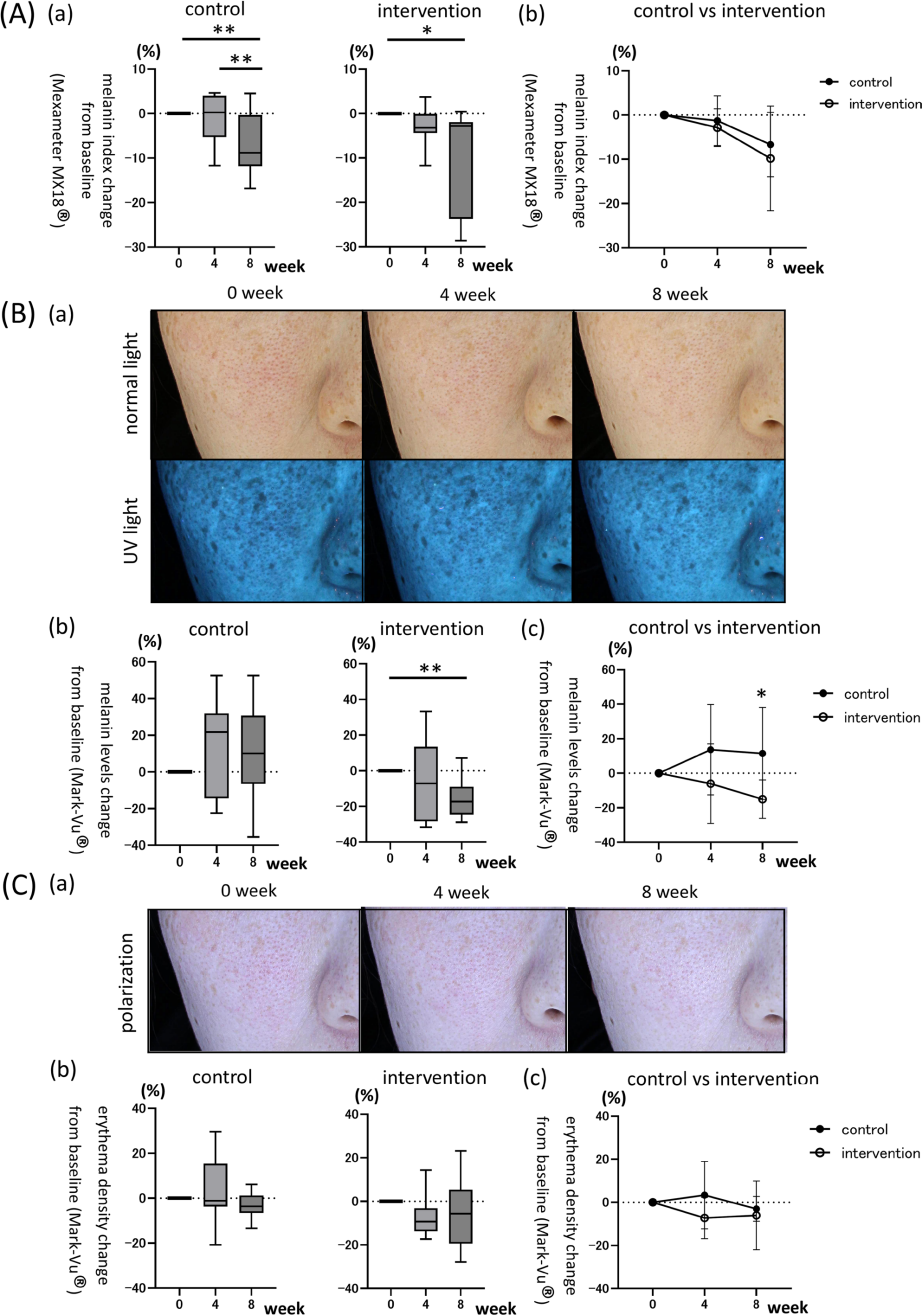
Changes in melanin and erythema. (A) Melanin levels measured using the Mexameter device. (a) Within‐group comparisons at baseline, week 4, and week 8 in both the control and intervention groups (*n* = 9 per group). Box plots represent the median (line within the box), the interquartile range (IQR; box), and the whiskers indicate the minimum and maximum values. *p < 0.05, **p < 0.01 versus baseline, week 4, or week 8. (b) Between‐group comparisons at week 4 and week 8 (*n* = 9 per group). Error bars indicate SD. *p < 0.05 for comparisons between control and intervention groups. (B) Melanin levels assessed using the Mark‐Vu system. (a) Representative photographs of the intervention group at baseline, week 4, and week 8 under normal and ultraviolet light conditions (*n* = 9 per group). (b) Within‐group comparisons at baseline, week 4, and week 8 in both the control and intervention groups (*n* = 9 per group). Box plots represent the median (line within the box), the interquartile range (IQR; box), and the whiskers indicate the minimum and maximum values. *p < 0.05, **p < 0.01 vs. baseline, week 4, or week 8. (c) Between‐group comparisons at week 4 and week 8 (*n* = 9 per group). Error bars indicate SD. *p < 0.05 for comparisons between control and intervention groups. (C) Erythema measurements using the Mexameter and Mark‐Vu systems. (a) Representative polarized‐light images of the intervention group at baseline, week 4, and week 8 using the Mark‐Vu system. (b) Within‐group comparisons of erythema index measured by the Mexameter at baseline, week 4, and week 8 (*n* = 9 per group). Box plots represent the median (line within the box), the interquartile range (IQR; box), and the whiskers indicate the minimum and maximum values. (c) Between‐group comparisons of erythema index measured by the Mexameter at week 4 and week 8 (*n* = 9 per group). Error bars indicate SD.

Melanin levels were assessed using the Mexameter MX18, which emits two distinct wavelengths (660 and 880 nm) and quantifies the amount of light absorbed by the skin [20, 22]. In the control group, a significant reduction in melanin levels was observed at week 8 compared to baseline (Figure 3A (a), left panel, −6.68% ± 7.05%, *p* < 0.01). A notable difference was also observed between weeks 4 and 8 (Figure 3A (a), left panel, −1.29% ± 5.46% [4‐week] vs. −6.68% ± 7.05% [8‐week], *p* < 0.01). In the intervention group, melanin levels also showed a significant decrease at week 8 compared with baseline (Figure 3A (a), right panel, −9.82% ± 11.83%, *p* < 0.05). Between‐group comparisons demonstrated non‐inferiority of the intervention group at both time points, although there was no significant difference (Figure 3A (b)). During the study, skin photographs were taken using the Mark‐Vu system under both normal and UV light [23, 24], and representative images of the intervention group are presented in Figure 3B (a), illustrating a visible reduction in melanin pigmentation. The corresponding quantitative analysis of melanin levels, based on these images and measured by the Mark‐Vu system, is shown in Figure 3B (b) and (c), which indicates no significant changes from baseline in the control group (Figure 3B (b), left panel). In contrast, the intervention group demonstrated a significant reduction in melanin at week 8 (Figure 3B (b), right panel, −15.01% ± 11.10%, *p* < 0.01). A significant between‐group difference was observed at week 8, with greater melanin reduction in the intervention group (Figure 3B (c), 8‐week: 11.44% ± 26.67% [control] vs. −15.01% ± 11.10% [intervention], *p* < 0.05). Erythema density was also measured using the Mexameter MX18 [22], which emits wavelengths of 568 and 660 nm and quantifies absorbed light (Figure 3C). Time‐dependent image changes were documented using polarization mode (Figure 3C (a)), which shows representative images from the intervention group. Neither group showed significant changes in erythema at weeks 4 or 8 (Figure 3C (b)), and no significant differences were found between groups (Figure 3C (c)).

### 3.5 Improvement of the Skin Scores Based on Participant Questionnaire in the Intervention Group

**FIGURE 4:**
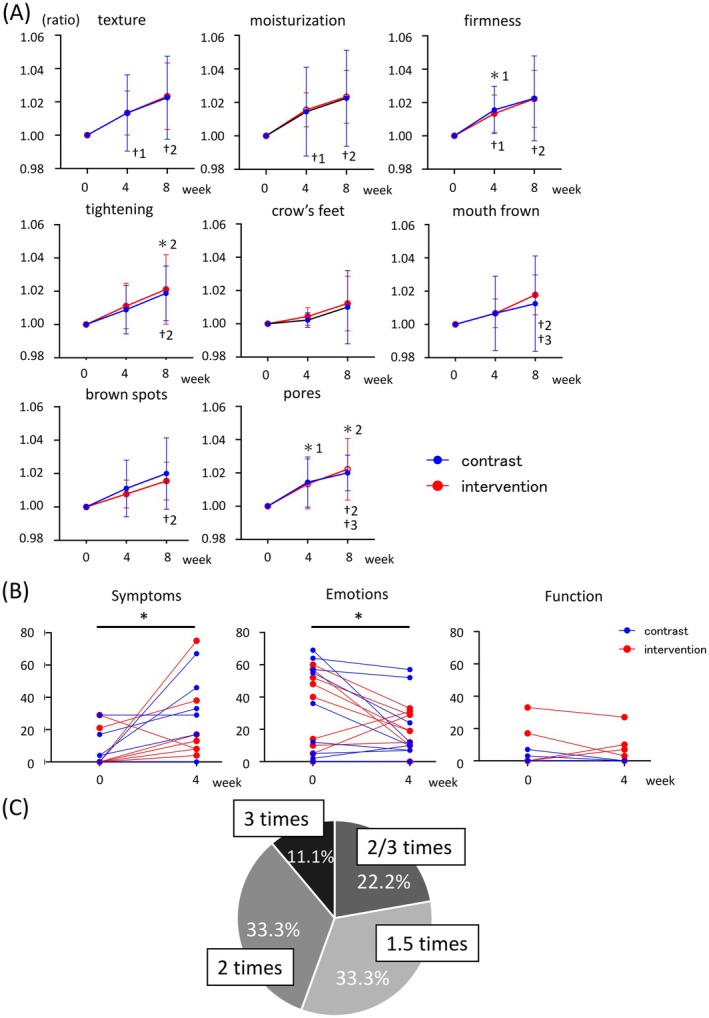
Participant questionnaire results. (A) Subjective evaluations of skin condition at baseline, week 4, and week 8 (*n* = 9 per group). Error bars indicate SD. *1: *p* < 0.05, comparison between baseline and week 4 in the control group. *2: *p* < 0.05, comparison between baseline and week 8 in the control group. †1: *p* < 0.05, comparison between baseline and week 4 in the intervention group. †2: *p* < 0.05, comparison between baseline and week 8 in the intervention group. †3: *p* < 0.05, comparison between week 4 and week 8 in the control group. (B) Change in Skindex‐16 scores between baseline and week 4 in the control and intervention groups (*n* = 9 per group). *p < 0.05, comparison between baseline and week 4. (C) Changes in product usage behavior following the instructional intervention in the intervention group (*n* = 9).

A subsequent analysis of the participants' self‐assessment questionnaires revealed improvements in skin texture and moisturization scores in both the control and intervention groups compared to baseline (Figure 4A, the first row). For skin texture, no statistically significant differences were observed in the control group throughout the study period. In contrast, the intervention group showed significant increases at both weeks 4 and 8 (Figure 4A, the first row, baseline vs. 1.01 ± 0.01 [4‐week], *p* < 0.05; baseline vs. 1.02 ± 0.02 [8‐week], *p* < 0.05). A similar trend was observed for moisturizing. While no significant changes were detected in the control group, the intervention group exhibited significant increases at both time points (Figure 4A, the first row, baseline vs. 1.02 ± 0.01 [4‐week], *p* < 0.01; baseline vs. 1.02 ± 0.02 [8‐week], *p* < 0.01). Skin firmness and tightening also increased in both groups (Figure 4A, the second row). In the control group, skin firmness significantly increased at week 4 (Figure 4A, the second row, baseline vs. 1.02 ± 0.01 [4‐week], *p* < 0.05), whereas in the intervention group, significant improvements were observed at both weeks 4 and 8 (Figure 4A, the second row, baseline vs. 1.01 ± 0.01 [4‐week], *p* < 0.05; baseline vs. 1.02 ± 0.02 [8 week], *p* < 0.05). Skin tightening scores increased significantly at week 8 in both groups (Figure 4A, the second row, control: baseline vs. 1.02 ± 0.02 [8‐week], *p* < 0.05; intervention: baseline vs. 1.02 ± 0.02 [8‐week], *p* < 0.05). Scores for crow's feet, mouth frown, brown spots, and pores also increased in both groups (Figure 4A, the third row). A significant improvement of mouth frown was observed only in the intervention group at week 8, both relative to baseline and week 4 (Figure 4A, the third row, 1.01 ± 0.01 [4‐week] vs. 1.02 ± 0.01 [8‐week], *p* < 0.05; baseline vs. 1.02 ± 0.01 [8‐week], *p* < 0.01). For brown spots, a significant improvement was detected in the intervention group at week 8 (Figure 4A, the third row, baseline vs. 1.02 ± 0.01 [8‐week], *p* < 0.01). Regarding pores, the control group showed significant increases at both weeks 4 and 8 (Figure 4A, the third row, baseline vs. 1.01 ± 0.02 [4‐week], *p* < 0.05; baseline vs. 1.02 ± 0.01 [8‐week], *p* < 0.01), while the intervention group exhibited a significant increase at week 8 relative to both baseline and week 4 (Figure 4A, the third row, baseline vs. 1.02 ± 0.02 [8‐week], *p* < 0.05; 1.01 ± 0.02 [4‐week] vs. 1.02 ± 0.02 [8‐week], *p* < 0.05).

The Skindex‐16, a validated dermatology‐specific quality of life instrument consisting of 16 questions assessing symptoms, emotions, and function [25], was used to evaluate changes in QOL at baseline and week 4 (Figure 4B). Across all participants, symptom scores increased (Figure 4B, left panel, 14.89% ± 23.97%, *p* < 0.05). In contrast, emotion scores decreased overall (Figure 4B, middle panel, −12.50% ± 21.50%, *p* < 0.05). However, no significant differences were found between the control and intervention groups in symptom, emotion, or function domains. To further explore behavioral changes, a follow‐up questionnaire was administered to assess the method and amount of product applied (Figure 4C). 78% of participants reported changes in their topical application technique following the instructional intervention. Additionally, approximately 80% of participants reported an increase in the amount of product used, with the most common increase being 1.5 to 2 times the amount previously applied.

### 3.6 Adverse Effects

44% of the participants reported experiencing some stimulation on at least one day; however, with the exception of two individuals, all participants completed the experiment without dropout (according to the daily logbook).

## 4 Discussion

This study focused on skin anti‐aging as a component of broader anti‐aging strategies. Specifically, it investigated the effectiveness of skincare and the impact of instructional interventions on skincare outcomes. Objective skin parameters were evaluated to assess the efficacy of appropriate instructional interventions. The skin care product used in this study was a microneedle‐containing cream formulated with hydroxypropyl chitosan, hydrolyzed collagen, *malpighia emarginata* (acerola) fruit extract, mannan, enantia chlorantha bark extract, lactobacillus/soymilk ferment filtrate, sodium hyaluronate, niacinamide, and centella asiatica extract, among other ingredients (Table S1). Various delivery methods are being developed to enhance the penetration of skin care products into the skin [26, 27]. One of these methods is microneedling, in which microneedles are generally 100–1000 μm in length, 8–250 μm in width, and 1–25 μm thick at the tip. They are composed of materials such as silicone, polymers, and metals [26, 27]. When microneedles penetrate the skin, they create microchannels that induce controlled skin injury while minimizing damage to the epidermis, thereby stimulating the wound healing response. The proliferation and migration of fibroblasts promote neovascularization and neocollagenesis. A fibronectin network is formed, providing a matrix for the deposition of type III collagen, which is eventually replaced by type I collagen [28]. Indeed, histological changes such as increased epidermal thickness and elevated levels of collagen and elastin fibers have been reported by Aust et al. [29].

Microarray analysis by Schmitt et al. identified an upregulation of genes associated with tissue remodeling and wound healing (e.g., *COL3A1*, *COL8A1*, *TIMP3*), epithelial proliferation and differentiation (*KRT13*, *IGF1*), immune cell recruitment (*CCL11*), and a member of the heat shock protein family (*HSPB6*) [30]. Additionally, Aust et al. demonstrated upregulation of TGF‐β3 [31]. Clinically, microneedle application has been reported to improve a variety of dermatologic conditions, including acne, pigmentation such as melasma, skin elasticity, and wrinkle formation [32, 33, 34, 35]. The microneedles used in this study were composed of a hydrophilic biopolymer derived from cancellous bone and are characterized by numerous micropores, which are believed to enhance ingredient penetration [18, 19]. The microneedles used in this study are not intended for use as medical devices; however, they are formulated within a topical cream, permitting safe and practical self‐application by the user. This product was selected based on the hypothesis that outcome differences between participants receiving skin care instructions and those not receiving instructions would be more discernible when using this microneedle formulation.

With aging, the skin's ability to retain moisture declines, resulting in sagging, wrinkles, and hyperpigmentation, which together contribute to a duller complexion [1, 2, 3]. In addition to photoprotection, a balanced diet, and adequate sleep, skin care is considered an essential component of anti‐aging strategies. AAD recommends continuous moisturization as a basic preventive measure [3]. Such age‐related changes in facial appearance can negatively affect social interactions, and interventions that improve these signs may contribute to enhanced quality of life (QOL) [4]. In this study, the primary endpoint was the intracorneal water content, an established indicator of moisturization efficacy. Wrinkles and pigmentation were also evaluated as representative aging parameters. The results indicated that application of the microneedle cream led to increased skin hydration and reduced wrinkles and pigmentation. These effects were further enhanced by the provision of skin care instruction. Stratum corneum water content and TEWL are key indices for assessing skin moisturization [7, 16, 20]. Intracorneal water content measures the amount of water present within the keratin, with increases indicating improved moisturization [20]. TEWL quantifies water loss through the epidermis, with reductions suggesting enhanced barrier function [16, 20]. In this study, intracorneal water content showed a significant increase throughout the entire study period in the intervention group (Figure 1A, middle panel (intervention): 14.06% ± 14.61% [4‐week], *p* < 0.05; 27.99% ± 14.18% [8‐week], *p* < 0.01). Intervention group showed a significant increase compared to the control group at 4‐week and 8‐week mark (Figure 1A, right panel, 4‐week: −0.98% ± 13.49% [control] vs. 14.06% ± 14.61% (intervention), *p* < 0.05; 8‐week: 13.52% ± 10.04% [control] vs. 27.99% ± 14.18% [intervention], *p* < 0.05). TEWL in the intervention group showed significant reductions compared to the control group at both weeks 4 and 8 (Figure 1B, right panel, 4‐week: 4.97% ± 15.15% [control] vs. −13.75% ± 17.43% [intervention], *p* < 0.05; 8‐week: −6.58% ± 7.87% [control] vs. −16.95% ± 11.87% [intervention], *p* < 0.05). These findings suggest that microneedle cream efficacy is enhanced by appropriate skin care instruction, thereby improving the moisturization. The microneedle cream contains several ingredients with potential moisturizing properties, including sodium hyaluronate, hydrolyzed collagen, niacinamide, hydroxypropyl chitosan, and lactobacillus/soymilk ferment filtrate. The observed effects suggest that these active compounds were more effectively delivered into the skin through microneedle‐facilitated permeation [36, 37, 38, 39, 40].

The viscoelasticity and elasticity of the skin were evaluated to assess wrinkle‐related biomechanical properties. The Cutometer DUAL MPA580 is a device commonly used to quantify viscoelasticity and elasticity [20, 21, 22]. Both the intervention and control groups exhibited significant within‐group improvements in overall skin elasticity. The intervention group showed earlier improvement at week 4 (15.75% ± 9.37%, *p* < 0.01), whereas the control group demonstrated significant improvement at week 8 (13.54% ± 13.24%, *p* < 0.05). Although no statistically significant differences were found between the two groups overall, the earlier improvement observed in the intervention group highlights the potential benefit of skincare instruction. For the specific elasticity parameters R5 and R7, no significant changes were observed at week 4 (Figure 2A, R5, left panel, control: 1.76% ± 11.33%, *p* = 0.89; middle panel, intervention: 8.87% ± 16.92%, *p* = 0.35; R7, left panel, control: −3.48% ± 10.36%, *p* = 0.59; middle panel, intervention: 7.92% ± 10.29%, *p* = 0.14). However, a significant increase was noted at week 8 compared with week 4 in the control group (Figure 2A, R5, left panel: 1.76% ± 11.33% [4‐week] vs. 20.48% ± 14.58% [8‐week], *p* < 0.01; R7, left panel: −3.48% ± 10.36% [4‐week] vs. 18.87% ± 12.66% [8‐week], *p* < 0.01). Notably, in R7, a significant between‐group difference was observed at week 4, in favor of the intervention group, further underscoring the impact of skin instruction. These findings suggest that microneedle cream improves skin viscoelasticity and elasticity, with enhanced efficacy when combined with appropriate skin care instruction. Moreover, incorporating microneedle cream with proper skin care guidance may effectively reduce wrinkle formation. To ascertain the efficacy of this approach in preventing future wrinkle development, we employed the Mark‐Vu wrinkle prediction model [23, 24]. A trend of reduction in future wrinkles was observed in both the intervention and control groups over time; a significant improvement was observed in the intervention group at week 8 compared to week 4 (Figure 2B, middle panel, 4‐week: 6.99% ± 25.44% vs. 8‐week: −12.12% ± 33.84%, *p* < 0.05). These results indicate that long‐term use of microneedle cream with appropriate skin care guidance may be effective in preventing future wrinkle development. Hydrolyzed collagen and sodium hyaluronate have been suggested to contribute to wrinkle prevention [37, 41], while niacinamide has been reported to smooth fine lines [38]. *Centella asiatica* extract has also been suggested to promote the synthesis of collagen and intracellular fibronectin [42]. These properties, in combination with the microneedles' own ability to stimulate dermal remodeling [34], may have contributed to the observed improvements in skin viscoelasticity and the prevention of future wrinkle formation.

Age spots are primarily recognized by visible differences in the distribution of melanin pigment in the skin [43, 44]. This study demonstrated that microneedle cream exerted a depigmenting effect. Both the intervention and control groups exhibited a reduction in melanin‐associated pigmentation, as assessed using the Mexameter MX18 [20, 22], which measures light absorption at wavelengths corresponding to melanin (Figure 3A). Additionally, Mark‐Vu imaging revealed a significant reduction in melanin levels at week 8, particularly in the intervention group (Figure 3B (b), right panel, −15.01% ± 11.10%, *p* < 0.01). A direct comparison between the intervention and control groups at week 8 further confirmed a statistically significant difference (Figure 3B (c), control: 11.44% ± 26.67% vs. intervention: −15.01% ± 11.10%), underscoring the importance of comprehensive skin care instructions. A notable factor contributing to the reduction in melanin pigmentation might be the niacinamide in the microneedle cream. Niacinamide has been documented to inhibit the transfer of melanin granules from melanocytes to keratinocytes [43, 45]. As previously described, microneedling has been reported to improve hyperpigmentation, including melasma [33, 34, 35]. It is therefore plausible that the observed reduction in melanin pigmentation in this study may have resulted from a combination of the effects of microneedling itself and the active ingredient niacinamide. Considering the potential for microneedle creams to induce irritation or contact dermatitis [26, 27], erythema incidence was carefully monitored. However, no significant changes were detected throughout the study period in either the control or intervention groups (Figure 3C (c), 4‐week: 3.37% ± 15.62% [control] vs. −7.24% ± 9.60% [intervention], *p* = 0.10; 8‐week: −3.01% ± 5.75% [control] vs. −6.04% ± 15.91% [intervention], *p* = 0.60). Furthermore, no clinical signs of inflammation—commonly associated with irritation from microneedle use—were observed in either group over the course of the study.

The skin condition questionnaire revealed that the participants were subjectively aware of improvements across all evaluated items (Figure 4A). Improvements in skin texture and hydration reflect enhanced moisturization, while increases in skin firmness and resilience correspond to improved elasticity. Furthermore, reductions in wrinkles around the eyes and mouth are also indicative of enhanced skin elasticity. Many participants also reported visible improvements in hyperpigmentation (spots) and pore appearance. These findings suggest that the anti‐aging effects observed through objective measurements were also perceived subjectively by the participants. Additionally, the Skindex‐16 emotional domain scores improved regardless of whether skincare instructions were provided, indicating a psychological benefit from cream application itself. However, the symptom domain scores worsened. This deterioration may be attributed to transient irritation or itching, which was reported by 44% of participants according to the daily logbook. On the other hand, as shown in Figure 3C, no significant changes in skin redness were observed in either the intervention or control groups throughout the 8‐week study period, indicating that the skincare intervention did not lead to any issues related to skin color tone. According to the questionnaire assessing changes in product usage following the instructional intervention, the majority of participants reported an increase in the amount of cream applied. Notably, most participants experienced a 1.5‐ to 2‐fold increase in the amount applied, suggesting that the observed skin improvements were influenced not only by the method of application, but also by the increased quantity of product used. In previous studies involving patients with atopic dermatitis, educational interventions led to clinical improvements, with increased use of topical agents cited as one of the contributing factors [8]. Similarly, in the present study, improvements in both the amount and method of product application may have facilitated more appropriate skin care behavior and enhanced the transdermal delivery of active ingredients via microneedles, thereby contributing to the observed intervention effects. While an increased application volume may raise concerns about potential adverse effects such as itching, stinging, or redness associated with microneedling, no such differences in skin index parameters were observed between the instruction and non‐instruction groups. These findings suggest that appropriate external guidance—including recommendations on optimal dosage—can enhance the efficacy of microneedle‐based interventions while minimizing the risk of side effects.

In this study, the daily application of microneedle cream over an 8‐week period led to significant improvements in skin moisturization and viscoelasticity, as well as a reduction in melanin pigmentation. Notably, the improvements in skin hydration and elasticity were significantly greater among participants who received skincare instructions. The participants' subjective evaluations were in agreement with the objective measurements, indicating that they perceived a visible improvement in their skin condition. Appropriate skincare instruction may help mitigate potential adverse effects associated with increased product usage. These findings underscore the importance of providing skincare education and suggest that healthcare professionals should adopt a more proactive role in promoting evidence‐based skincare practices.

## Author Contributions

This work was a collaborative effort. All authors discussed the results and contributed to the final manuscript. T.M., J.O., Y.O., R.K., T.F., A.Y., and T.Y. conducted the research. T.M., R.K., J.O., and T.Y. designed the study. J.O. provided essential reagents or tools. T.M., J.O., and T.Y. performed the data analysis. T.M. and T.Y. wrote the manuscript.

## Ethics Statement

This study was conducted in accordance with the ethical standards of the institutional and national research committee and with the 1964 Helsinki declaration and its later amendments or comparable ethical standards. Informed consent was obtained from all individual participants included in the study. All procedures performed in studies involving human participants were in accordance with the ethical standards of the University of Tokyo and with the 1964 Helsinki Declaration and its later amendments or comparable ethical standards. This study was approved by the Research Ethics Committee of the Faculty of Medicine of the University of Tokyo (approval 2023011NI). It is registered in UMIN‐CTR (UMIN000052080).

## Conflicts of Interest

Dr. Takashi Yamashita had an advisory agreement with YA‐MAN Ltd., Japan, a supplier of cosmetics, to provide expertise in dermatology as a dermatologist.

## Supporting information


**Table S1:** Active ingredients of this microneedle cream. This is the list of active ingredients in the microneedle cream used for the study. The concentrations of each ingredient were not available due to trade confidentiality.

**Table S2:** How to instruct skin care using microneedle cream by medical professionals. Medical professionals used the Japanese version of this document to explain the procedure to participants and also provided visual instructions using a video model.

## Data Availability

The data that support the findings of this study are available from the corresponding author upon reasonable request.
