# Supplementary material for: Facial Skin Care Instruction by Medical Professionals Using Microneedle Cream: The 8‐Week Prospective, Randomized, Single‐Blinded for Examiners Trial in Asians
Source: J Cosmet Dermatol. 2026 Feb 2;25(2):e70680. doi: 10.1111/jocd.70680 (PMC12865263; doi:10.1111/jocd.70680)
Supplement: Supplementary file 2 — Table S2: How to instruct skin care using microneedle cream by medical professionals. Medical professionals used the Japanese version of this document to explain the procedure to participants and also provided visual instructions using a video model. [file JOCD-25-e70680-s001.docx]

Supplementary table 2

How to apply:

1. Apply a pearl-sized amount of cream to your whole face, especially on areas where it feels dry.

2. Gently tap the area around your eyes and mouth to blend the cream.

3. For areas that are particularly dry, apply a bit more pearl-sized amount of cream in the same way.

4. Use the whole palm of your hand to blend the cream into your face.

Attention:

Check to see if there are any areas you forgot to apply cream.

Avoid rubbing too much or tapping too hard.

Be careful not to get the cream in your eyes.
